# Biological Influence of Nonswelling Microgels on Cartilage Induction of Mouse Adipose-Derived Stem Cells

**DOI:** 10.1155/2019/6508094

**Published:** 2019-10-13

**Authors:** Zheng Liu, Jun Wang

**Affiliations:** ^1^Department of Spine Surgery, Xiangya Hospital, Central South University, 87, Xiangya Road, Changsha 410008, Hunan, China; ^2^Hunan Engineering Laboratory of Advanced Artificial Osteo-Materials, Xiangya Hospital, Central South University, 87, Xiangya Road, Changsha 410008, Hunan, China

## Abstract

In cartilage tissue engineering, the target cells' functional performance depends on the biomaterials. However, it is difficult to develop an appropriate scaffold to differentiate mouse adipose-derived stem cells (mADSCs) into chondrocyte despite an increasing number of studies on biological scaffold materials. The purpose of this study was to create a novel scaffold for mADSC culture and chondrogenic differentiation with a new series of microgels based on polyethyleneimine (PEI), polyethylene glycol (PEG), and poly (L-lactic acid) (PLLA) and able to resist swelling with changes in temperature, pH, and polymer concentration. The biocompatibility and ability of the nonswelling microgels were then examined and served as scaffolds for cell culture and for cartilage differentiation. The results show that the new microgels are a novel biomaterial that both retains its nonswelling properties under various conditions and facilitates important scaffold functions such as cell adhesion, proliferation, and cartilage induction.

## 1. Introduction

Cartilage defect disease is a global health problem and surgical intervention remains its only therapeutic modality with a reparative effect [1–4]. Tissue engineering provides a unique treatment for cartilage reparation [5–7]. The operative usage of tissue engineering to repair cartilage disease requires the optimal combination of three key ingredients: seed cells, biological scaffolds, and growth factors [8–10]. This study was interested in the biological scaffolds. The ideal biological materials must be able to retain their biocompatibility [11–13] and provide a good biological environment for cell growth.

In tissue engineering, delivery platforms such as natural and synthetic nanogels, microgels, and hydrogels are coming out because of their excellent mechanical properties, degradation rates, tunable architectural features, biocompatibility, and capacity to deliver any cargos [14–19]. These systems have been applied to various biomedical usage so far, which includes: three-dimensional platforms (such as, microfluidic or array) to study in vitro cellular responses [20–22], cell delivery platforms which are capable of regulating paracrine responses to angiogenesis [23], peptide, and protein delivery vehicles [24, 25].

Microgels and hydrogels are cross-bonded hydrophilic networks of polymers that swell in water [26] and are widely used in regenerative medicines as implantable and injectable biomaterials [27], temporary scaffolds for cell culture, or as reservoirs for drug release [26, 28–30]. Although microgels are useful in many applications, their tendency to swell can cause several disadvantages, which include weakening the mechanical properties, damaging the neighboring tissues, compressing the nearby nerves, and even displacement from the implantation sites [31–34]. Several nonswelling microgels had previously been reported, but each experienced swelling when conditions such as temperature and pH were changed [35–41]. These early attempts at creating a nonswelling mycrogel relied on the balancing of the forces between those exerted due to the hydrophobic (shrinking) portion of the mycrogel and the hydrophilic (swelling) portion to attain a mycrogel that resisted swelling in water. The mycrogels reported here is a nonswelling mycrogel system that resists various changes in temperature, pH, and polymer concentration for usage in drug release and tissue engineering.

Unlike the previous nonswelling mycrogel systems, the one presented herein works by utilizing the limitations to put on swelling through a hyperbranched, crosslinked polyethyleneimine (PEI) network with hydrophobic poly (L-lactic acid) (PLLA) units incorporated. Relatively nontoxic low MW PEI was used in the procedure to address the critical cytotoxicity associated with high MW PEI [42]. Poly (ethylene glycol) (PEG) is the crosslinker in this system and was used to increase the hydrophilicity and biocompatibility of the polymer network, as had been previously done [43, 44].

In this study, the newly developed microgels (PEI, P3, P6, P12) were used to test if they could support mADSC attachment, proliferation, and their chondrogenic differentiation for cartilage tissue regeneration.

## 2. Materials and Methods

### 2.1. Fabrication of Hydrogels and Microgels

Four-armed poly(L-lactide) was obtained through ring opening polymerization (ROP) of LLA as reported in previous work [45, 46]. For a PLA sample with twelve repeats of each arm, 8.000 g of LLA monomer, 0.315 g initiator (pentaerythritol), and 18 *µ*L co-initiator of Sn(Oct)2 were added and mixed in a flask, placed under vacuum, then purified in a nitrogen atmosphere. This purification process was repeated three times. The reaction proceeded at 110°C for two days. 32 mL DCM was used to dissolve the product and the solid product PLA12 was obtained from precipitation in 320 mL hexane/diethyl ether (*v*/*v* = 95 : 5). Samples prepared from PLA with 6 and 3 repeats in each arm resulted in PLA6 and PLA3, respectively. The procedure to produce prepolymers PLA3 and PLA6 was the same as for PLA12. The molecular weight (MW) of each four-armed PLA prepolymer was calculated by the ratio of the integrals of medium CH at 5.14 to the end CH at 4.33 of PLA. The MW of PLA3, PLA6, and PLA12 are 1117 g/mol, 1772 g/mol, and 3799 g/mol, separately. Then the PLA end group was modified with an aldehyde group by conjugating FA at the terminal −OH of PLA with a polyester according to previous work [45]. Briefly, PLA12, PLA, FA, DCC, and DMAP were combined in a molar ratio of 1 : 6 : 6 : 6 before being dissolved in DCM. The reaction was carried out at room temperature for 3 days. The turbid solution was filtered and the clear filtrate was precipitated in the hexane/diethyl mixture ether (*v*/*v* = 95 : 5) three times to obtain PLA12-FA. Third, PLA12-FA prepolymers were modified with PEI to enhance hydrophilicity. DCM was used to dissolve the PLA12-FA and PEI separately. Later, the low MW PEI solution (MW 1800 Da) was added dropwise to the PLA12-FA solution, and stirred overnight. This solution was precipitated in hexane to obtain PLA12-FA-PEI prepolymers. Then the prepolymers were dissolved in PBS and then the crosslinker PEGDE was added. The final concentration of prepolymer and crosslinker was set as 16 wt%. The solutions were vortexed and kept at 37°C overnight. The hydrogel samples were labeled P0, P3, P6, and P12 corresponding to the number of PLA repeat units. The hydrogel without PLA was also labeled as PEI. In the following test,we used hydrogel samples to test its swelling behaviors. P0 was chosen as control for the swelling test, as it contains no PLLA and P6 was chosen as representative sample of the hydrogels.

After we synthesised the hydrogel material, we explored their potential for their use as microgels for cell scaffolding. The microgels used in biological study are made from the hydrogels,briefly, a 1 ml 30 wt% mixture of prepolymer and crosslinker was dropped into 20 mL mineral oil/span 80 (*v* : *v* = 20 : 0.4) and stirred at room temperature overnight. The product was centrifuged at a speed of 5000 rpm and washed with hexane then with distilled water three times. The product was suspended in distilled water and lyophilized for later use in the biological study.

### 2.2. Measurement of the Degree of Swelling and Degradation

Hydrogels were prepared in tubular molds with an inner diameter of 7.2 mm. The hydrogel samples were cut into small cylinders and immersed in 10 mL buffer solution (pH = 5, 7.4, and 9) and put in a 37°C incubator or cooled at 4°C. The degree of swelling (Q) was calculated from the change in weight (*Q* = *mt*/*m*0), where mt is the weight at each time point and m0 is the initial weight of the hydrogel. The remaining mass (*Rt*/*R*0) was calculated. The test was done in triplicate for each microgel sample.

### 2.3. Isolation and Culture of Mouse Adipose-Derived Stem Cells (ADSCs)

All the animal experiment procedures were approved by animal ethics committee of central south university. Adipose tissues were collected by needle biopsy from mice. Then the tissue samples were washed extensively by PBS which contained 5% P/S (penicillin/streptomycin). After the remaining was removed, the tissue was put in a sterile cell culture dish containing 0.075% collagenase Type I, which was dissolved in phosphate-buffered saline that contained 2% penicillin/streptomycin. Then the tissue sample was minced with scalpels, pipetted several times with a 25 or 50 ml pipette and put in the incubator for half an hour at 37°C and 5% CO_2_. After incubation, 5 ml of a-MEM that contained 20% FBS(fetal bovine serum, Atlanta Biological, Atlanta, GA) was added to neutralize the collagenase Type I. The tissue sample was pipetted up and down to break the masses. After the disintegration, a 50 ml centrifuge tube was used to collect the tissue sample. Then the sample was centrifuged at the speed of 2000 rpm for 5 min and shaken violently two times. After the spinning, all the supernatant was aspirated without disturbing the cells. The cell pellets were resuspended with no more than 3 ml of culture medium (a-MEM contained 20% FBS, 1% L-glutamine and 1% P/S). When they 70–80% blended together, the cells were subpassaged and the ADSCs before P5/passage 5 were used in the biology studies.

### 2.4. The Seeding of mADSC and Chondrogenic Induction

**Figure 1:**
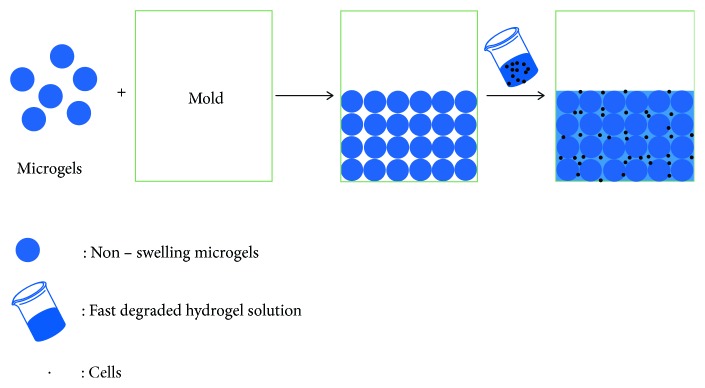
Microgels serve as scaffolds for cell culture. Microgels were mixed with a cell suspension in a 96-well model. The microgels supported the cells in culture medium and the space between the microgels allows cells to infiltrate into the scaffold.

Before the cell seeding, the PEI, P3, P6, and P12 microgels were rinsed in ethanol and freeze-dried overnight. Then 400 *μ*L of mADSCs suspension (87.5∗10^4^/mL) (DMEM medium was used to suspend the cells, considered as fast degraded hydrogel solution) was seeded into 1 well of microgel (6 mg/well) and put in the incubator at 37°C with 5% CO_2_ for 2 h (Figure 1). Here, both microgels and fast degraded hydrogel solution (e.g., DMEM medium) can be liquid form and they can be injected into the desire site. The mADSCs were seeded into the following five separate groups: (1) the microgel PEI group, 3D negative control (PEI); (2) the microgel P3 group (P3); (3) the microgel P6 group (P6); (4) the microgel P12 group (P12); and (5) the control group: 2D negative control (TCP). And then the cell/microgel complex was cultured in cartilage induction medium (High Glucose-DMEM that contained L- proline (40 *μ*g/ml), ascorbic acid (0.1 mM), dexamethasone (0.1 *μ*M), recombinant human TGF-*β*1 (10 ng/ml, PeproTech Inc., NJ, USA), 1 × ITS Premix (BD Biosciences), 1% FBS, and 1% P/S. After 14 days of induction, the cell/microgel complex was washed with PBS and then put in the 4% cold formaldehyde for following tests like Safranin O staining, RT-PCR and Western Blot to evaluate chondrogenesis. For gene and protein expression, TCP group was added as a control group without any material added and also acted as traditional chondrogenic treatment group.

### 2.5. Proliferation

In order to test the material cytotoxicity, mADSCs were seeded on the PEI, P3, P6, and P12 microgels. After being sterilized by ethanol and UV light, ADSCs were then seeded on 6 mg of the microgels in 200 *µ*L culture medium (5∗10^4^ cells/well). CellTiter 96 ® AQueous One Solution Cell Proliferation Assay was used to evaluate the cell proliferation after having been cultured for 1, 3, 5, and 7 days, respectively. At each point in time, each well was added with 40 *µ*L of assay medium. After having been incubated for 2 hours at 37°C, a new 96-well plate was used to collect 150* μ*L medium from each sample and the absorbance at 490 nm was measured with a Varioskan Flash multimode reader (Thermo Scientific, Wyman Street Waltham, MA).

### 2.6. Scanning Electron Microscopy (SEM)

After being observed by a stereo microscope, the blank microgel was covered with gold for SEM observation. Two weeks after induction, the cell/microgel complex was put in 2.5% phosphate-buffered glutaraldehyde (Sigma-Aldrich, St. Louis, MO) overnight in the refrigerator at 4°C, and follow by fixation of 1% osmium tetroxide (Sigma-Aldrich) for 1 h. Then these samples were washed 3 times with PBS, and a graded series of ethanol was used to dehydrate them. After this, the sample was left to dry in hexamethyldisilazane (HMDS, Sigma-Aldrich) [47]. After the procedures above, the samples were coated with gold and imaged at 15 kV with a JEOL-7800FLV scanning electron microscope (JEOL, USA) to observe cell adhering, spreading, and extracellular matrix secretion in the microgels.

### 2.7. Safranin O Staining

After two weeks after cartilage induction, cell/microgel complex was washed with PBS and put in 4% formaldehyde overnight. After two other washes with PBS, the complex was stained with Safranin O solution.

### 2.8. Real-Time PCR (Real-Time Polymerase Chain Reaction)

First, a disposable plastic pestle (Fisher Scientific, Pittsburg, PA) was used to crush the cell/microgel complex, and then Trizol reagent (Life Technologies Corporation) was applied to extract the RNA from the complex. After measuring the RNA concentration according to the absorbance at 260 nm, the SuperScript II cDNA Synthesis kit (Invitrogen) was used to synthesize the complementary DNA (cDNA) in accordance with the manufacturer's protocol. Real-time polymerase chain reaction was performed using Syber green PCR Master Mix (Applied Biosystems) with predesigned primers for Sox9 (Forward: GACTTCCGCGACG-TGGAC, Reverse: GTTGGGCGGCAGGTACTG) and Collagen type II (Forward: CCGTGGTGAGGCTGGTC, Reverse: GCACCAGGTTGG-CCATCA). ABI 7500 Real-time PCR System (Applied Biosystems) was applied to perform the reactions. The gene performance was normalized by housekeeping gene 18S performance (Forward: TAGAGGGACAAGTGGCGTTC, Reverse: CGCTGAGCCAGTCAGT-GT).

### 2.9. Western Blot

All the proteins were extracted from cultured cell/microgel complexes and the protein concentration was measured by a BCA Protein Quantification Kit (Pierce, USA). Then the protein was separated by 10% sodium dodecyl sulfate-polyacrylamide gel electrophoresis followed by the transfer to a PVDF membrane (Millipore, USA). In order to block the membranes, TBS-T (Tris-buffered saline-tween) and 1% BSA were used. The blocking was done at room temperature for 1 h. Primary antibody anti-collagen 2A1 (1 : 500 in TBS-T, Santa Cruz Biotechnology) or anti-*β*-actin antibody (1 : 1000 in TBS-T, Cell Signaling) was used for membrane incubation at 4°C overnight and then the membrane was incubated in a horseradish peroxidase (HRP)-labeled anti-mouse or anti-rabbit secondary IgG (1 : 5000 in TBS-T, Pierce) at room temperature for 2 h. An ECL Prime Western Blotting detection reagent (GE Healthcare) was applied to detect the immunoreactive bands on the membrane and then the membrane was exposed to X-Posure films (Thermo Scientific) for 5–10 min.

### 2.10. Statistical Analysis

The data in this study were shown in an average value ± standard deviation form. The experiments were done in triplicate to ensure reproducibility. Prism 6 (Graphpad, Inc.) software and Image J software were used to analyze the data in this study. Especially, statistical significance was determined by *t*-tests. *P* < 0.05 was considered statistically significant.

## 3. Results

### 3.1. Swelling Test

**Figure 2:**
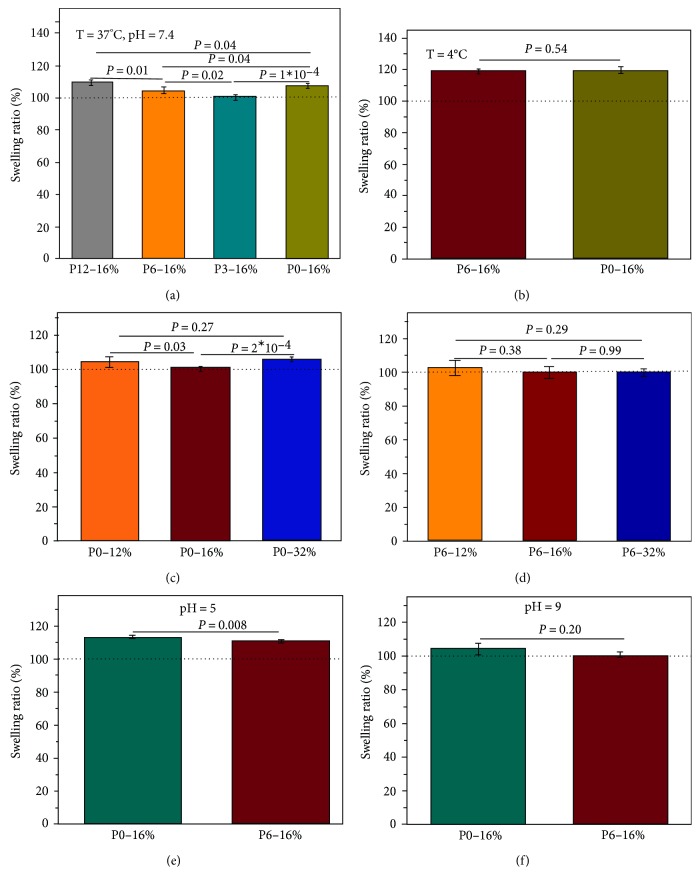
Swelling behaviors of the hydrogels in various conditions. A swelling ratio of 100% corresponds to no swelling. Generally, hydrogels with median amounts of PLLA (P3, P6) swelled less than the PEI-based hydrogel (P0). Swelling ratios were lowest at 37°C and at pH = 9, but nonswelling was maintained at pH = 7.4 for every hydrogel tested. (a) Swelling ratio of P0-P12 hydrogels were measured in PBS (pH = 7.4) at 37°C; (b) swelling ratios of P6 and P0 hydrogels in PBS at 4°C; (c, d) swelling ratios of P0 and P6 at varying concentrations, respectively; (e, f) swelling ratios of P6 and P0 at 37°C in pH = 5 and pH = 9 PBS solution, respectively.

The swelling ratios of hydrogels P0–P12 at 16 wt% are shown in Figure 2(a). Briefly, the swelling ratio was calculated as the ratio of the sample mass at the end of the experiment (5d) to initial sample mass before immersion in PBS and converted to a percent; a swelling ratio of 100% corresponds to no mass change. All samples demonstrated a low swelling ratio, consistently below 110% at physiological conditions (*T* = 37°C, pH = 7.4), indicating the hydrogels' ability to retain their nonswelling properties throughout the experiment (Figure 2(a)).

We next tested the hydrogels' ability to resist swelling at various conditions by changing the temperature, concentration, and pH. P0 was chosen as the positive control for the remainder of the experiments, as it contains no PLLA and P6 was chosen as representative sample for the remainder of experiments. Both P0 and P6 hydrogels swell more at 4°C than at 37°C (Figure 2(b)); P6 swells more than P0 due to the hydrophobic PLLA segment becoming more hydrated at lower temperatures [48]. We next tested the swelling ratios of P0 and P6 hydrogels with polymer concentrations of 12  and 32 wt% compared to the original 16 wt%, and found that they maintained their nonswelling property for each concentration tested (Figures 2(c) and 2(d)). P0 hydrogels at 12  and 32 wt% swelled significantly more than hydrogels containing 16 wt% polymer (Figure 2(c)). While lower weight percent hydrogels (P0-12% and P6-12%) retained their nonswelling property, they were found to be weaker than the corresponding high concentration hydrogels. For these reasons, hydrogels of 16 wt% polymer were used in the remaining of experiments except where otherwise noted. We also tested the swelling behavior at different pH values (Figures 2(e) and 2(f)). The highest swelling ratio for both P0 and P6 occurred in pH = 5, with median swelling at pH = 7.4, and the lowest swelling ratio at pH = 9. This is as expected, as the amine groups on the PEI segments absorb more protons in the lower pH solution, thereby increasing their positive charge, leading to increased electrostatic repulsion between the polymer chains in the hydrogel [49]. This is also evident as the P0 hydrogels, containing more PEI, swelled significantly more than P6 hydrogels at pH = 5. Even in low pH medium, P6 and P0 hydrogels each showed a swelling ratio of less than 120%.

### 3.2. Proliferation

**Figure 3:**
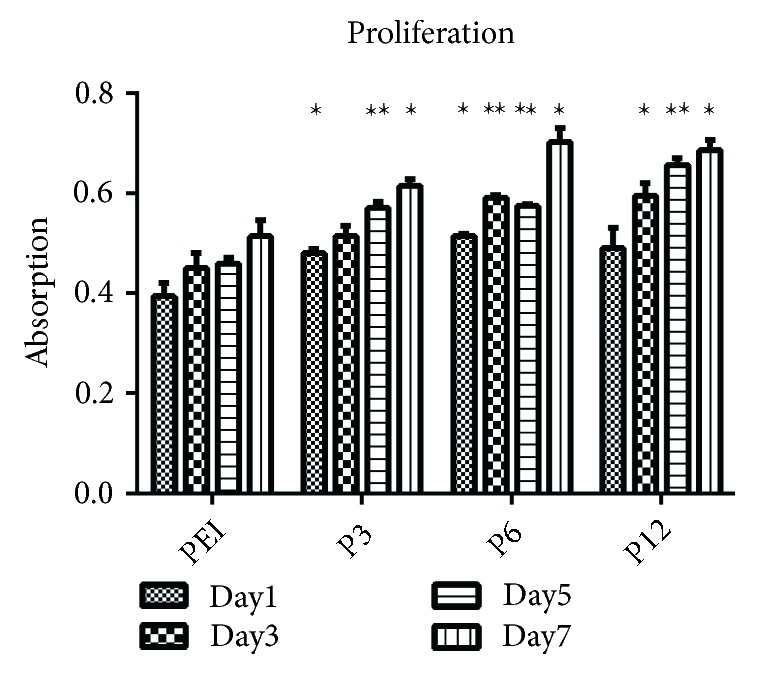
The proliferation of ADSCs in the microgels. Data represented mean ± SD; Asterisks indicate statistical significance compared to PEI groups at each point-in-time (^∗^*P* < 0.05; ^∗∗^*P* < 0.01). The error bars indicate the standard deviations of three independent assays (*n* = 3).

We next prepared microgels, crosslinked hydrogel particles used as 3D constructs to support cells during culture [14, 50–52]. CellTiter-Blue Assay was used to assess how the microgels supported cell proliferation. As the results showed, the number of cells in each microgel group was basically the same on days 1, 3, 5, and 7 of culture, but still had several differences. The absorbance of the PEI group was less than that of the P3 group and P6 group at day 1 (*P* < 0.05 for P3 versus PEI; *P* < 0.05 for P6 versus PEI); P6 group and P12 group demonstrated higher absorbance than PEI group at day 3 (*P* < 0.01 for P6 versus PEI; *P* < 0.05 for P12 versus PEI); all of these three groups had higher absorbance than PEI groups at day 5 and day 7(*P* < 0.01 for P3 versus PEI, P6 versus PEI, P12 versus PEI at day 5; *P* < 0.05 for P3 versus PEI, P6 versus PEI, P12 versus PEI at day 7). Furthermore, all the groups shared similar growth trends, which indicated that none of the microgels influenced cell proliferation negatively (Figure 3).

### 3.3. Scanning Electron Microscopy (SEM) Observation

**Figure 4:**
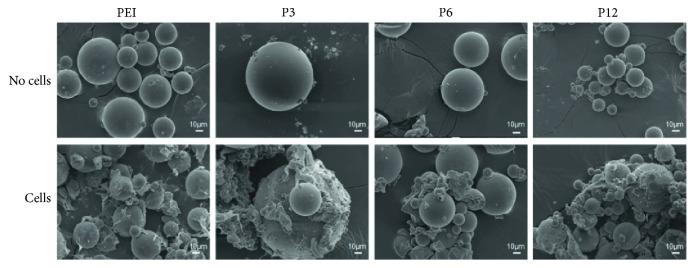
SEM views of microgels and cell attachment on the scaffolds. SEM observation showed that the microgels have a varying sphere structure of smooth surface. There was space between any two microgel spheres. The mouse adipose-derived stem cells (ADSCs) retained and spread well both on the surfaces of scaffolds and between the microgels.

Microstructure properties such as pore distribution, shapes, and porous inner structure significantly influence cell attachment, infiltration, cell growth and functions in tissue engineering [51–53]. The morphology of cell/microgel is shown in Figure 4, where Microgel P3 had sizes that ranged from 50 *μ*m to 100 *μ*m, Microgel P6 had sizes that ranged from 10 *μ*m to 50 *μ*m, and Microgel P12 had sizes that ranged from 5 *μ*m to 50 *μ*m. The pictures showed that there was space between the microgels so that cells could be attached to, grow, and interact with each other. The effects of differentiation may have something to do with it.

### 3.4. Microgels Supporting mADSC Chondrogenic Differentiation

**Figure 5:**
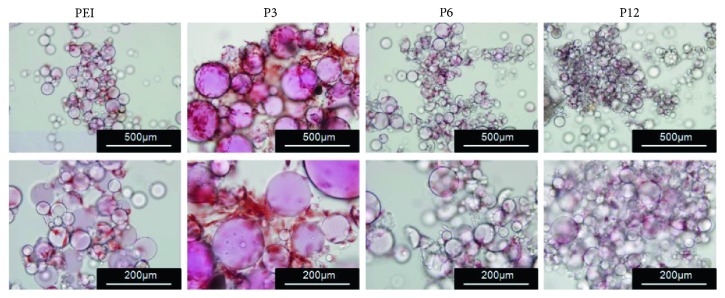
The chondrogenesis induction by the microgels was evaluated by Safranin O staining. Two weeks after chondrogenic induction, Safranin O staining was done on the cell/microgel constructs to detect sulfated glycosaminoglycans (sGAG) performance. Bar scale = 500 *μ*m (upper) or 200 *μ*m (lower).

Next, several biochemical tests were used to answer the question whether the microgels can promote the chondrogenesis of the seeded mADSCs in the microgels. The Safranin O staining results showed that, compared with the control group, microgel P3 directed mADSCs into chondrocyte-like cells as confirmed by positive Safranin O staining to show excellent performance of sGAG after 14 days of culture (Figure 5) while the other microgels showed less positive staining.

**Figure 6:**
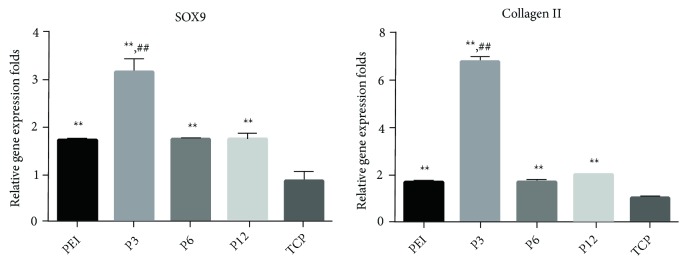
The induction of chondrogenesis by the microgels was measured by RT-PCR. The expression of the housekeeping gene 18S was used to normalize all the other gene expression. Data represented mean ± SD; Asterisks indicate statistical significance compared to TCP or PEI group (∗∗Compared to TCP group, *P* < 0.01; ##Compared to PEI group, *P* < 0.01). The error bars indicate the standard deviations of three independent assays (*n* = 3).

RT-PCR was used to study the changes in gene performance. The performance of the classic marker SOX9 improved in mADSCs cultured in P3 microgel (*P* < 0.01), P6 microgel (*P* < 0.01), P12 microgel (*P* < 0.01) and PEI microgel (*P* < 0.01), with better performance on plates coated with P3 microgel compared to P6 microgel (*P* < 0.01), P12 microgel (*P* < 0.01) and PEI microgel groups (*P* < 0.01). Compared with PEI microgel group, P6 and P12 microgel groups showed little differences in SOX9 performance (*P* > 0.05). There was a significant improvement in Collage II mRNA performance in the P3, P6, P12, and PEI microgel groups (*P* < 0.01), with better performance of P3 microgel group compared to P6 microgel (*P* < 0.01), P12 microgel (*P* < 0.01), and PEI microgel groups (*P* < 0.01). In addition, Collagen II performance in the P6 and P12 microgel groups had no significant differences in comparison with those in the PEI microgel group (Figure 6).

**Figure 7:**
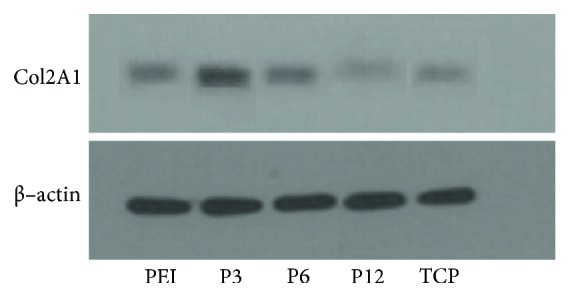
Western blot of collagen type II and *β*-actin in microgels (*n* = 3).

**Figure 8:**
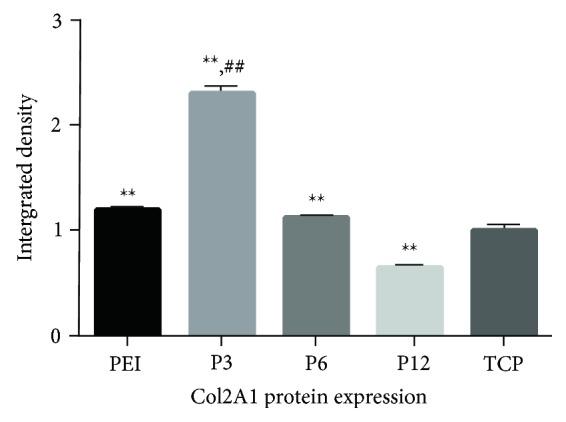
Densitometric data of western blotting in Col2A1 (∗∗Compared to TCP group, *P* < 0.01; ##Compared to PEI group, *P* < 0.01). The error bars indicate the standard deviations of three independent assays. *n* = 3).

The protein performance of collagen 2A1 on Day 14 was analyzed by western blotting and quantified by image J software. The microgel P3 group exhibited the best performance of collagen 2A1 levels compared to the TCP group and PEI group on Day 14. The differences in collagen 2A1 performance between the other microgel groups were ambiguous (Figures 7 and 8).

## 4. Discussion

Cartilage tissue engineering has the possibility to be good for the entire adult population [54–56]. Nevertheless, cartilage extracellular matrix is only secreted by chondrocyte—a terminal differentiated cell type. Chondrocyte is hard to isolate from tissue and has very limited expansion ability. Hence, stem cells which can differentiate chondrogenic lineages are highly considered as cell sources for cartilage regeneration. Although there were significant advances in recent stem cell biology, inducing stem cells into the desired direction still remains a daunting challenge. Therefore, scaffolds, another component of tissue engineering, could be developed to imitate the microenvironment and then control the differentiation directions of stem cell [57–61]. This study was a good attempt that the novel microgels were applied to enhance the chondrogenesis of mADSCs and thereby promote cartilage regeneration.

For cartilage tissue engineering, a material which can be injected is more preferable than the material which can only be implanted because of the small and irregular shape of cartilage defects. Porous microspheres are found as injectable cell vectors for tissue regeneration [62–65], but microgels had not been used in previous technologies. Hydrogels with linear prepolymer will swell in aqueous solution as the flexible chains extend and absorb water, while the hydrogel with hyperbranched prepolymer will not swell due to difficulty in deforming the short chain branches. Linear prepolymers can form a loose hydrogel at low concentration, while hyperbranched prepolymers with compact chains form a very weak network or cannot form a network because the short branches cannot extend far enough to interact with each other. However, once the concentration of hyperbranched prepolymers is high enough to form a hydrogel (12 wt% or above), the compact structure gives the hydrogel its nonswelling property. Presented here are a series of novel, nonswelling microgels that retain their nonswelling property with changes in pH, temperature, and polymer concentration. These novel injectable microgels were used to repair cartilage, and the small and irregular shaped cartilage defects can be easily filled up.

Spaces between the microgel spheres mimicked the fibrous architectures of extracellualr matrix and were shown to promote mADSC growth and different induction when they were compared with nonnanofibrous materials [57, 66, 67]. Consistently, in comparison with the control microgel group, the cell attachment, growth, and their chondrogenesis were significantly improved by the biomimetic nanofibrous features and the porous structures of the spaces between microgels. Because the average cell diameter in cell suspension is around 10 *µ*m, a microgel with diameter under 100 *µ*m was designed to promote the cell infiltration into the spaces between every two microgels.

Accordingly, more cells were attached to the microgel P3 surface than to the other microgels. Not only the cell attachment and growth was improved, but also the requisite cell to cell interactions were facilitated by the highly interconnected spaces between the microgels to enhance the collagen II expressions and chondrocyte formation.

In previous studies, various scaffolds (i.e., fibrous titanium meshes, porous ceramics, or collagen sponge) were used to seed mADSCs, but they always formed a tissue that resembled a connective tissue more than a cartilage-like one [68] and demonstrated the significant challenges of controlling the chondrogenesis of mADSCs. In the present study, once the mechanism responsible for the nonswelling nature of the hydrogels was investigated, we explored their potential for their use as microgels for cell scaffolding. Some classic chondrogenic markers were examined in mADSC cultures in microgel P3, microgel P6, microgel P12, and microgel PEI. GAG is the main ECM of cartilage and widely used as a classic marker of chondrogenesis. The Safranin O result showed that the GAG content in the microgel P3 group was significantly higher than that in either the microgel P6 or the microgel P12 group at the end of 2 weeks of induction. SOX9 and Collagen II genes are the traditional markers in chondrogenesis. These two genes showed better performance in the microgel P3 group than in the other groups. Similarly, collagen 2A1 protein is widely used as a typical marker in chondrogenesis. The Collagen 2A1 protein performance level in the microgel P3 group was significantly higher than that in either the microgel P6 or the microgel P12 group at the end of 2 weeks of induction. All of these *in vitro* results indicated that the microgel P3 enhanced the chondrogenic differentiation of mADSCs in comparison with the other microgels. Taken together, the data indicated that microgels made from our novel nonswelling hydrogels can support cell growth and chondrogenic differentiation of mADSCs in conditions in which they retain their nonswelling property.

What is more, microgels stand for a new kind of injectable material which introduces microscale interconnected porosity by robust achievements of imperfect self-assembly and anneal of individual building blocks. Both clinical and *in vitro* requirements for biomaterials can be solved by this general approach. The microgels can perfectly fill tissue defects and provide interconnected microporosity between the microgels at the same time. These features of the microgels could change clinical wound treatment by using the microgel/cell construct as a plastic cartilage substitute and as a artificial replacement to the transplantation application. Furthermore, the microgels' injectability will allow them to be used in any wound despite the sizes and shapes. This feature is very important especially in the situation when functional tissues are regenerated.

The microgels present here has their unique microporosity and injectability. And it will affect the future development of cartilage regeneration *in vivo* and cartilage tissue creation.

## 5. Conclusion

In conclusion, the microgels were fabricated successfully to imitate the natural microenvironment for cartilage tissue regeneration, which were beneficial for the ADSC attachment, proliferation, and chondrogenesis. The results demonstrated that microgel is a promising injectable cell carrier with high clinical potential for cartilage reparation. The cell/microgel system for cartilage tissue engineering and the beneficial effects of this scaffold need to be explored further.

## References

[1] Silver F. H., Glasgold A. I. (1995). Cartilage wound healing. An overview. *Otolaryngologic Clinics of North America*.

[2] Convery F. R., Akeson W. H., Keown G. H. (1972). The repair of large osteochondral defects. an experimental study in horses. *Clinical Orthopaedics and Related Research*.

[3] Wei X., Gao J., Messner K. (1997). Maturation-dependent repair of untreated osteochondral defects in the rabbit knee joint. *Journal of Biomedical Materials Research*.

[4] Deng Z. H., Li Y. S., Gao X., Lei G. H., Huard J. (2018). Bone morphogenetic proteins for articular cartilage regeneration. *Osteoarthritis and Cartilage*.

[5] Wakitani S., Goto T., Pineda S. J., Young R. G., Mansour J. M., Caplan A. I. (1994). Mesenchymal cell-based repair of large, full-thickness defects of articular cartilage. *The Journal of bone and joint surgery American*.

[6] Langer R., Vacanti J. P. (1993). Tissue engineering. *Science*.

[7] Liao J., Wang S., Chen J., Xie H., Zhou J. (2017). Progress in application of 3D bioprinting in cartilage regeneration and reconstruction for tissue engineering. *Journal of Central South University Medical Sciences*.

[8] Hardingham T., Tew S., Murdoch A. (2002). Tissue engineering: chondrocytes and cartilage. *Arthritis Research*.

[9] Frenkel S. R., Di Cesare P. E. (2004). Scaffolds for articular cartilage repair. *Annals of Biomedical Engineering*.

[10] Athanasiou K. A., Shah A. R., Hernandez R. J., LeBaron R. G. (2001). Basic science of articular cartilage repair. *Clinics in Sports Medicine*.

[11] VandeVord P. J., Matthew H. W., DeSilva S. P., Mayton L., Wu B., Wooley P. H. (2002). Evaluation of the biocompatibility of a chitosan scaffold in mice. *Journal of biomedical materials research*.

[12] Hutmacher D. W. (2000). Scaffolds in tissue engineering bone and cartilage. *Biomaterials*.

[13] Qu F., Zhang Y., Rasooly A., Yang M. (2014). Electrochemical biosensing platform using hydrogel prepared from ferrocene modified amino acid as highly efficient immobilization matrix. *Analytical Chemistry*.

[14] Jiang Y., Chen J., Deng C., Suuronen E. J., Zhong Z. (2014). Click hydrogels, microgels and nanogels: emerging platforms for drug delivery and tissue engineering. *Biomaterials*.

[15] Zhang H., Zhai Y., Wang J., Zhai G. (2016). New progress and prospects: The application of nanogel in drug delivery. *Materials Science & Engineering C, Materials for Biological Applications*.

[16] Molina M., Asadian-Birjand M., Balach J., Bergueiro J., Miceli E., Calderon M. (2015). Stimuli-responsive nanogel composites and their application in nanomedicine. *Chemical Society Reviews*.

[17] Wang H., Heilshorn S. C. (2015). Adaptable hydrogel networks with reversible linkages for tissue engineering. *Advanced Materials*.

[18] Wu Z., Zou X., Yang L., Lin S., Fan J., Yang B. (2014). Thermosensitive hydrogel used in dual drug delivery system with paclitaxel-loaded micelles for in situ treatment of lung cancer. *Colloids and surfaces B, Biointerfaces*.

[19] Zhou M., Sun Z., Shen C., Li Z., Zhang Y., Yang M. (2013). Application of hydrogel prepared from ferrocene functionalized amino acid in the design of novel electrochemical immunosensing platform. *Biosensors & Bioelectronics*.

[20] Siltanen C., Yaghoobi M., Haque A., You J., Lowen J., Soleimani M. (2016). Microfluidic fabrication of bioactive microgels for rapid formation and enhanced differentiation of stem cell spheroids. *Acta Biomaterialia*.

[21] Li Y., Chen P., Wang Y., Yan S., Feng X., Du W. (2016). Rapid Assembly of Heterogeneous 3D Cell Microenvironments in a Microgel Array. *Advanced Materials*.

[22] Vu L. T., Jain G., Veres B. D., Rajagopalan P. (2015). Cell migration on planar and three-dimensional matrices: a hydrogel-based perspective. *Tissue Engineering Part B, Reviews*.

[23] Thomas D., Fontana G., Chen X., Sanz-Nogues C., Zeugolis D. I., Dockery P. (2014). A shape-controlled tuneable microgel platform to modulate angiogenic paracrine responses in stem cells. *Biomaterials*.

[24] Bysell H., Mansson R., Hansson P., Malmsten M. (2011). Microgels and microcapsules in peptide and protein drug delivery. *Advanced Drug Delivery Reviews*.

[25] Liu J., Liu H., Kang H., Donovan M., Zhu Z., Tan W. (2012). Aptamer-incorporated hydrogels for visual detection, controlled drug release, and targeted cancer therapy. *Analytical and Bioanalytical Chemistry*.

[26] Hoffman A. S. (2012). Hydrogels for biomedical applications. *Advanced Drug Delivery Reviews*.

[27] Zheng Y., Liang Y., Zhang D., Sun X., Liang L., Li J. (2018). Gelatin-Based Hydrogels Blended with Gellan as an Injectable Wound Dressing. *ACS Omega*.

[28] Peppas N. A., Hilt J. Z., Khademhosseini A., Langer R. (2006). Hydrogels in biology and medicine: from molecular principles to bionanotechnology. *Advanced materials*.

[29] Slaughter B. V., Khurshid S. S., Fisher O. Z., Khademhosseini A., Peppas N. A. (2009). Hydrogels in Regenerative Medicine. *Advanced Materials*.

[30] Zhao J., Zhao X., Guo B., Ma P. X. (2014). Multifunctional interpenetrating polymer network hydrogels based on methacrylated alginate for the delivery of small molecule drugs and sustained release of protein. *Biomacromolecules*.

[31] Kamata H., Li X., Ui C., Sakai T. (2015). Design of hydrogels for biomedical applications. Advanced healthcare. *Materials*.

[32] Thavarajah D., De Lacy P., Hussain R., Redfern R. M. (2010). Postoperative cervical cord compression induced by hydrogel (DuraSeal): a possible complication. *Spine*.

[33] Sl B., Smyth M. D. (2007). Hydrogel-induced cervicomedullary compression after posterior fossa decompression for Chiari malformation: case report. Journal of Neurosurgery. *Pediatrics*.

[34] Mulder M., Crosier J., Dunn R. (2009). Cauda equina compression by hydrogel dural sealant after a laminotomy and discectomy: case report. *Spine*.

[35] Kamata H., Kushiro K., Takai M., Ui Chung, Sakai T. (2016). Non-osmotic hydrogels: a rational strategy for safely degradable hydrogels. *Angewandte Chemie*.

[36] Kamata H., Akagi Y., Kayasuga-Kariya Y., Chung U-i, Sakai T. (2014). “Nonswellable” hydrogel without mechanical hysteresis. *Science*.

[37] Truong V. X., Ablett M. P., Richardson S. M., Hoyland J. A., Dove A. P. (2015). Simultaneous orthogonal dual-click approach to tough, in-situ-forming hydrogels for cell encapsulation. *Journal of the American Chemical Society*.

[38] Zhang X.-Z., Yang Y.-Y., Wang F.-J., Chung T.-S. (2002). Thermosensitive Poly (N-isopropylacrylamide-co-acrylic acid) hydrogels with expanded network structures and improved oscillating swelling− deswelling properties. *Langmuir*.

[39] Hu C.-H., Zhang X.-Z., Zhang L., Xu X.-D., Zhuo R.-X. (2009). Temperature-and pH-sensitive hydrogels to immobilize heparin-modified PEI/DNA complexes for sustained gene delivery. *Journal of Materials Chemistry*.

[40] Barrett D. G., Bushnell G. G., Messersmith P. B. (2013). Mechanically robust, negative-swelling, mussel-inspired tissue adhesives. *Advanced Healthcare Materials*.

[41] Liu J., Gu T., Shan S., Kang S. H., Weaver J. C., Bertoldi K. (2016). Harnessing buckling to design architected materials that exhibit effective negative swelling. *Advanced Materials*.

[42] Abebe D. G., Kandil R., Kraus T., Elsayed M., Fujiwara T., Merkel O. M. (2016). Biodegradable three-layered micelles and injectable hydrogels. *Methods in Molecular Biology*.

[43] Teng L., Nie W., Zhou Y., Song L., Chen P. (2015). Synthesis and characterization of star-shaped PLLA with sorbitol as core and its microspheres application in controlled drug release. *Journal of Applied Polymer Science*.

[44] Daniele M. A., Adams A. A., Naciri J., North S. H., Ligler F. S. (2014). Interpenetrating networks based on gelatin methacrylamide and PEG formed using concurrent thiol click chemistries for hydrogel tissue engineering scaffolds. *Biomaterials*.

[45] Xie M., Wang L., Guo B., Wang Z., Chen Y. E., Ma P. X. (2015). Ductile electroactive biodegradable hyperbranched polylactide copolymers enhancing myoblast differentiation. *Biomaterials*.

[46] Guo B., Finne-Wistrand A., Albertsson A.-C. (2010). Molecular architecture of electroactive and biodegradable copolymers composed of polylactide and carboxyl-capped aniline trimer. *Biomacromolecules*.

[47] Liu Q., Tian S., Zhao C., Chen X., Lei I., Wang Z. (2015). Porous nanofibrous poly(L-lactic acid) scaffolds supporting cardiovascular progenitor cells for cardiac tissue engineering. *Acta Biomaterialia*.

[48] De las Heras Alarcón C., Pennadam S., Alexander C. (2005). Stimuli responsive polymers for biomedical applications. *Chemical Society Reviews*.

[49] Quesada-Pérez M., Maroto-Centeno J. A., Forcada J., Hidalgo-Alvarez R. (2011). Gel swelling theories: the classical formalism and recent approaches. *Soft Matter*.

[50] Griffin D. R., Weaver W. M., Scumpia P. O., Di Carlo D., Segura T. (2015). Accelerated wound healing by injectable microporous gel scaffolds assembled from annealed building blocks. *Nature materials*.

[51] Engler A. J., Sen S., Sweeney H. L., Discher D. E. (2006). Matrix elasticity directs stem cell lineage specification. *Cell*.

[52] Hirata I., Nomura Y., Ito M., Shimazu A., Okazaki M. (2007). Acceleration of bone formation with BMP2 in frame-reinforced carbonate apatite-collagen sponge scaffolds. *Journal of Artificial Organs*.

[53] Hutmacher D. W., Schantz J. T., Lam C. X., Tan K. C., Lim T. C. (2007). State of the art and future directions of scaffold-based bone engineering from a biomaterials perspective. *Journal of Tissue Engineering and Regenerative Medicine*.

[54] Macfarlane G. J., Thomas E., Croft P. R., Papageorgiou A. C., Jayson M. I., Silman A. J. (1999). Predictors of early improvement in low back pain amongst consulters to general practice: the influence of pre-morbid and episode-related factors. *Pain*.

[55] Fukuta S., Miyamoto K., Suzuki K., Maehara H., Inoue T., Hara A. (2011). Abundance of calpain and aggrecan-cleavage products of calpain in degenerated human intervertebral discs. *Osteoarthritis and Cartilage*.

[56] Rutges J. P., Duit R. A., Kummer J. A., Oner F. C., van Rijen M. H., Verbout A. J. (2010). Hypertrophic differentiation and calcification during intervertebral disc degeneration. *Osteoarthritis and Cartilage*.

[57] Wang J., Ma H., Jin X., Hu J., Liu X., Ni L. (2011). The effect of scaffold architecture on odontogenic differentiation of human dental pulp stem cells. *Biomaterials*.

[58] Ma P. X. (2008). Biomimetic materials for tissue engineering. *Advanced Drug Delivery Reviews*.

[59] Wang J., Liu X., Jin X., Ma H., Hu J., Ni L. (2010). The odontogenic differentiation of human dental pulp stem cells on nanofibrous poly(L-lactic acid) scaffolds in vitro and in vivo. *Acta Biomaterialia*.

[60] Smith L. A., Liu X., Ma P. X. (2008). Tissue engineering with nano-fibrous scaffolds. *Soft Matter*.

[61] Smith L. A., Ma P. X. (2004). Nano-fibrous scaffolds for tissue engineering. *Colloids and Surfaces B, Biointerfaces*.

[62] Fang J., Zhang Y., Yan S., Liu Z., He S., Cui L. (2014). Poly(L-glutamic acid)/chitosan polyelectrolyte complex porous microspheres as cell microcarriers for cartilage regeneration. *Acta Biomaterialia*.

[63] Ayen W. Y., Garkhal K., Kumar N. (2011). Doxorubicin-loaded (PEG)(3)-PLA nanopolymersomes: effect of solvents and process parameters on formulation development and in vitro study. *Molecular Pharmaceutics*.

[64] Kim T. K., Yoon J. J., Lee D. S., Park T. G. (2006). Gas foamed open porous biodegradable polymeric microspheres. *Biomaterials*.

[65] Guo L., Shi Y., Guo L., Zhang Q., Tian J., Zhu Y. (2012). Preparation and characterization of a titanium bonding porcelain. *Materials Science & Engineering C*.

[66] Qu T., Liu X. (2013). Nano-Structured Gelatin/Bioactive Glass Hybrid Scaffolds for the Enhancement of Odontogenic Differentiation of Human Dental Pulp Stem Cells. *Journal of Materials Chemistry B, *.

[67] Yang X., Yang F., Walboomers X. F., Bian Z., Fan M., Jansen J. A. (2010). The performance of dental pulp stem cells on nanofibrous PCL/gelatin/nHA scaffolds. *Journal of Biomedical Materials Research Part A*.

[68] Zhang W., Walboomers X. F., van Kuppevelt T. H., Daamen W. F., Bian Z., Jansen J. A. (2006). The performance of human dental pulp stem cells on different three-dimensional scaffold materials. *Biomaterials*.

